# P02.121. Psychological outcomes of a mind body program for successful aging

**DOI:** 10.1186/1472-6882-12-S1-P177

**Published:** 2012-06-12

**Authors:** M Scult, J Takahashi, A Webster, J Denninger, D Mehta

**Affiliations:** 1 Harvard Medical School, Boston, USA; 2 Benson-Henry Institute for Mind Body Medicine at Massachusetts General Hospital, Boston, USA

## Purpose

The biopsychosocial model of successful aging is aimed at developing a sense of well-being, high self-assessed quality of life, and a sense of personal fulfillment even in the context of illness and disability. The purpose of this study was to explore key outcomes of a new Successful Aging Mind Body program. We hypothesized the program would increase self-efficacy, which would lead to improved feelings of well-being.

## Methods

Sixteen patients completed assessments. The average age of participants was 75 (range: 66-91). The program consisted of weekly 90-minute sessions for nine weeks. Topics included a range of psychological and physical exercises including mindfulness and relaxation training. For measures, we used the Coping Self-Efficacy Scale (CSES) and Philadelphia Geriatric Center Morale Scale (PGCMS). We performed paired t-tests on pre/post data, and used the Wilcoxon signed rank test for the conservative validation.

## Results

We found that both the CSES and PGCMS increased among completers of the Successful Aging intervention (pre- to post-intervention change: CSES, 27.6±26.6, p=0.001; PGCMS 1.3±2.6, p=0.06), although the change for PGCMS was not quite significant. In addition, we found a moderate correlation between the pre- to post-intervention changes in CSES and PGCMS (r=0.4, p=0.14), although the association was not significant. The CSES change pre-post was still significant (p<.05) after a sensitivity analysis.

## Conclusion

We found significant changes in self-efficacy after the intervention and an increase in morale with a trend towards significance. A moderate correlation was found between CSES and PGCMS, but the correlation was not significant. These results support the hypothesis that participants can develop greater self-efficacy as a result of a mind body program for successful aging. Future research is needed to explore the relationship between self-efficacy and objective health outcomes.

